# Gingival Augmentation in the Thin Phenotype Using Injectable Platelet-Rich Fibrin and Microneedling

**DOI:** 10.7759/cureus.40435

**Published:** 2023-06-14

**Authors:** Rajnandini Adhikary, Prerna Mohan, Amit Wadhawan, Prashant Tyagi

**Affiliations:** 1 Department of Periodontology, Shree Bankey Bihari Dental College and Research Centre, Ghaziabad, IND

**Keywords:** phenotype, injectable platelet-rich fibrin, microneedling, gingival thickness, platelet-rich fibrin

## Abstract

Objective

This study set out to examine the results of microneedling (MN) with and without injectable platelet-rich fibrin (i-PRF) in treating individuals with thin periodontal phenotypes, specifically focusing on the effects on gingival thickness (GT) and keratinized tissue width (KTW).

Materials and methods

This was a split-mouth study involving 32 healthy patients with 64 sites having thin phenotypes classified into two groups. On the one side, i-PRF was injected (Group A), while on the other side (Group B), MN along with i-PRF was used at intervals of 10 days for a month. GT and KTW were measured at baseline, three months, and six months. The parameters were compared intra and intergroup-wise at baseline, three months, and six months using the IBM SPSS Statistics software version 24 (IBM Corp., Armonk, NY) and the chi-square test.

Results

The findings revealed that when comparing Group A, which received just i-PRF, to Group B, which had i-PRF in conjunction with MN, there was a statistically significant increase in GT in Group B.

Conclusions

This is a novel method in that it does not need surgical innervations to expand the gingiva in breadth or thickness. Although MN's neoangiogenesis impact contributed to thicker gingiva, i-PRF showed a greater capacity for the release of several growth factors.

## Introduction

Currently, dentistry practice is significantly influenced by aesthetics in a way that combines form and function. The phenotypic characteristics of the bone and soft tissues that make up the periodontium are described as the periodontal phenotype [1]. In 2017, experts gathered for the International Workshop on the Taxonomy of Periodontal and Peri-Implantation Diseases and Conditions, and a novel method was officially approved. It was made up of both the gingival phenotype (gum volume in three dimensions) and the bone morphotype (bone morphology). Measurements of the former group include keratinized tissue width (KTW) and gingival thickness (GT), whereas those of the latter group include buccal bone plate thickness (BBPT) [2]. The recession of the gingiva is more common in those who have a thin phenotype [3]. Microneedling (MN) or percutaneous collagen induction therapy creates microinjuries resulting in minimal superficial bleeding, and a cascade of wound healing is formed from which various growth factors are released [4]. Some people refer to MN as percutaneous collagen induction therapy (PCIT-MN) [5]. Platelet-rich fibrin is a unique kind of fibrin meshwork that serves as a reservoir for platelet-derived cytokines, growth factors, and cells. This fibrin network has the potential to function as a degradable membrane. This method was devised by centrifuging blood samples in tubes free of anticoagulants and activators [6]. Injectable platelet-rich fibrin (i-PRF) is a biomaterial made from blood that is a second-generation, completely autologous, three-dimensional fibrin meshwork comprising platelets and their growth factors, and it comprises collagen type 1 and lymphocytes [7]. After 10 days, i-PRF was shown to deliver growth factors at a higher rate than PRP, epidermal growth factor (EGF), insulin-like growth factor-1 (IGF-1), and several forms of platelet-derived growth factor (PDGF) [8].

The goal of this research is to compare the results of using i-PRF alone versus those of using i-PRF in conjunction with MN on thickening the gingival phenotype and the breadth of keratinized tissue in those who have a thin gingival phenotype.

## Materials and methods

This study included 64 locations in 32 individuals with the thin gingival phenotype and was conducted in the Department of Periodontology at Shree Bankey Bihari Dental College and Research Centre in Masuri, Ghaziabad. The study design employed was a prospective randomized single-blinded clinical research. In this study, we applied topical anesthetic gel to the patient's mandibular anterior region before each GT measurement and i-PRF-MN application. Hence patients were blinded as to which study treatment was performed on which sites.

The inclusion criteria were as follows: patients with a thin gingival biotype in the lower anterior between the ages of 18 and 34 years; nonsmokers; those who had a plaque index (PI) score of 0 or 1; those in whom bleeding on probing was not present; patients with GT of less than 0.8 mm in mandibular anterior teeth; and those with no malocclusions, crowding, or fillings missing or supernumerary in the mandibular anterior teeth. The exclusion criteria were as follows: patients with active orthodontic treatment, any previous periodontal surgery, any systemic diseases, use of blood thinners, use of any drugs that might lead to gingival enlargement, mucogingival stress, and bruxism.

**Figure 1:**
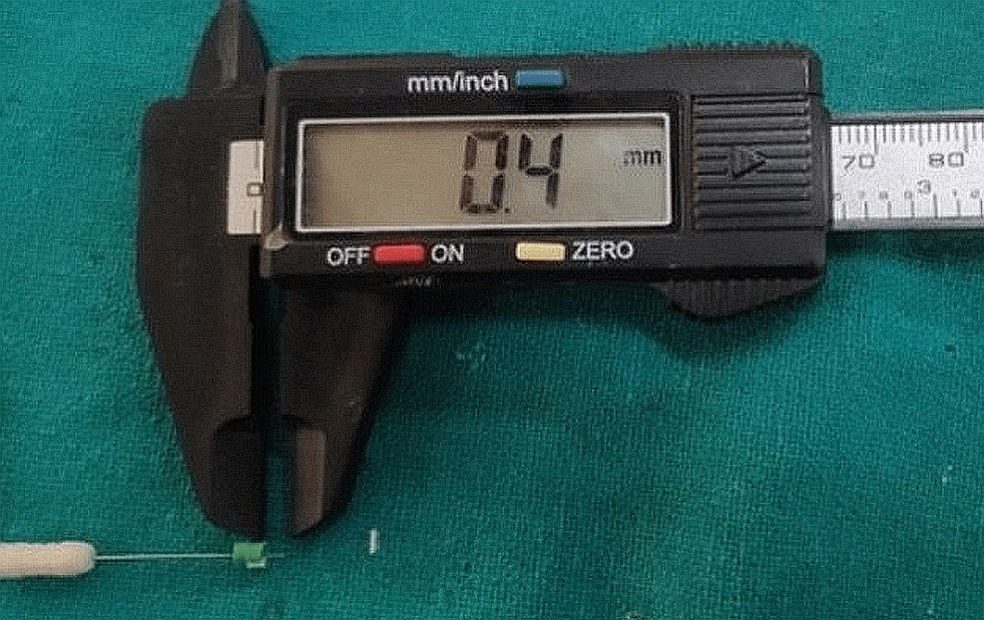
Vernier caliper to measure the gingival thickness

In this study, we measured the KTW and GT of the lower anterior. The measurement of KTW was done in the following manner: at first, the mucogingival junction was demonstrated using Lugol’s iodine solution. With the help of a periodontal probe with a silicon disc, the distance from the free gingival margin (the lower end of the silicone disc) to the mucogingival junction was measured through the vestibular midpoint. The measurement of GT was done with the help of a No. 15 endodontic spreader, which was placed in the center of a 3 mm-diameter silicone disc. The spreader was inserted perpendicularly from the vestibular midpoint at 1.5 mm apical of the gingival margin through the soft tissues until a hard surface was felt. The silicon disc was laid in tight contact with the soft tissue surface. The GT measurement was made 1.5 mm apical to the gingival margin, as the gingival margin overlapped with the coronal border of the silicone disc at that time. The penetration depth between the silicone disc and the spreader tip was measured with the help of a vernier caliper (Figure 1).

**Figure 2:**
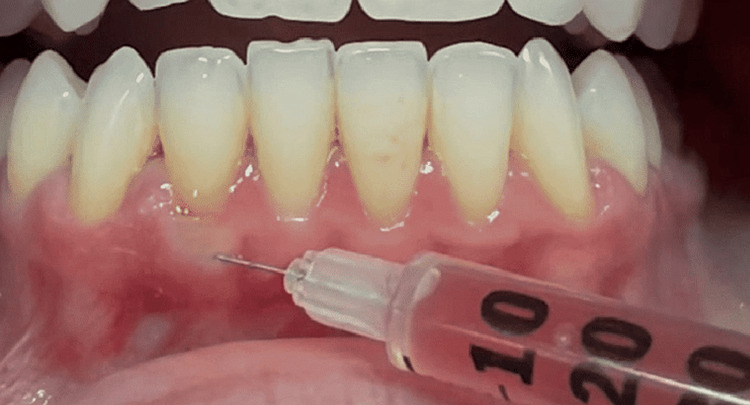
i-PRF is injected into the site i-PRF: injectable platelet-rich fibrin

A 20-ml injector was used on each patient to take blood from a vein. It was centrifuged for three minutes at room temperature at 700 revolutions per minute after being divided into two 10-ml i-PRF tubes with no anticoagulant. The collected i-PRF was then transferred to dental injectors holding 2.5 cc. While injecting i-PRF, we utilized a dental injector needle with a 27-gauge needle. The 33-gauge lancet needles were placed vertically into the tissue until they hit the bone (Figure 2).

**Figure 3:**
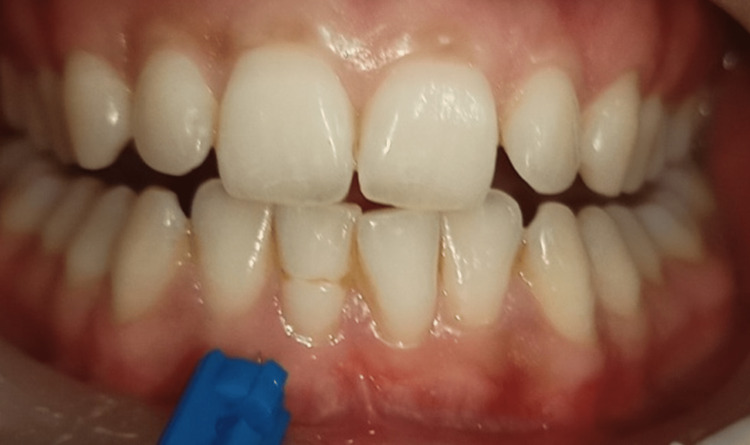
Preoperative microneedling in the gingiva

**Figure 4:**
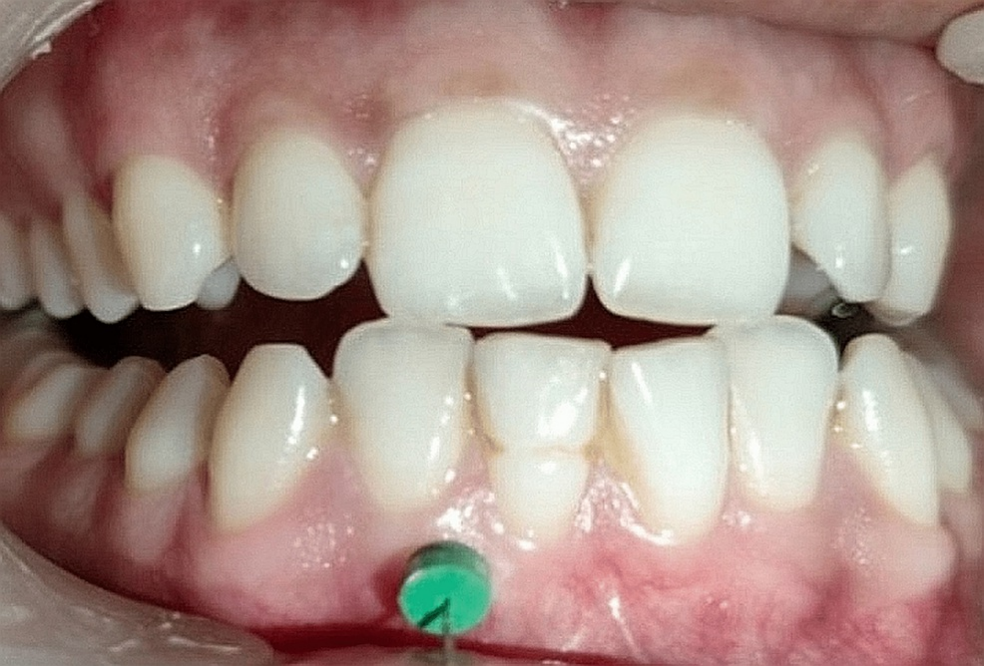
Postoperative measurements

As part of the MN treatment, a lancet was used to puncture the keratinized gingiva from the central tooth proximal to the distal gum line. One side of the front portion of the mandible received just i-PRF injections (Group A), whereas the other side received MN plus i-PRF injections (Group B). Both operations focused on the tip of the mucogingival junction of the alveolar mucosa. Before taking measurements and performing surgery, a local anesthetic gel was applied to the patient's mandibular anterior region. MN and the i-PRF technique were performed on patients four times, each separated by 10 days (Figures 3, 4).

The data thus obtained were statistically analyzed. The parameters were compared intra and intergroup-wise at baseline, three months, and six months using the IBM SPSS Statistics software version 24 (IBM Corp., Armonk, NY).

## Results

The results obtained showed a statistically significant increase in GT in Group B, which received i-PRF in combination with MN when compared with Group A, which received only i-PRF. There was also an increase in KTW, but it was not statistically significant in inter-group comparisons. When the same groups were compared again after six months, it was found that both GT and KTW had increased.

The mean KTW at baseline, three months, and six months, and the mean difference between baseline and at three months and baseline and at six months were compared between i-PRF alone (control) and i-PRF with MN using the unpaired t-test. There was no significant difference in mean KTW at baseline, three months, and six months, baseline to three months, and baseline to six months between i-PRF alone (control) and i-PRF with MN (Table 1).

**Table 1:** Comparison of keratinized tissue width between the groups i-PRF: injectable platelet-rich fibrin

Keratinized tissue width	i-PRF (control)		i-PRF with microneedling		Mean difference	t-test value	P-value
	Mean	Standard deviation	Mean	Standard deviation			
Baseline	3.26	0.63	3.29	0.59	-0.03	-0.205	0.838
3 months	3.89	0.78	3.96	0.87	-0.07	-1.722	0.515
6 months	3.97	0.72	4.03	0.98	-0.06	-1.992	0.398
Baseline to 3 months	-0.73	0.83	-0.77	0.78	0.04	1.984	0.507
Baseline to 6 months	-0.81	0.76	-0.84	1.08	0.03	4.559	0.603

The mean GT at baseline, three months, and six months, and the mean difference between baseline and three months and baseline and at six months were compared between i-PRF alone (control) and i-PRF with MN using the unpaired t-test. The mean GT at six months and baseline was significantly higher in i-PRF with MN compared to i-PRF alone (control) (Table 2).

**Table 2:** Comparison of gingival thickness between the groups i-PRF: injectable platelet-rich fibrin

Gingival thickness	i-PRF (control)		i-PRF with microneedling		Mean difference	t-test value	P-value
	Mean	Standard deviation	Mean	Standard deviation			
Baseline	0.57	0.19	0.62	0.21	-0.05	-1.045	0.300
3 months	0.64	0.20	0.74	0.23	-0.10	-1.692	0.124
6 months	0.69	0.20	0.87	0.22	-0.18	-2.962	0.035
Baseline to 3 months	-0.08	0.08	-0.12	0.08	0.04	1.014	0.099
Baseline to 6 months	-0.12	0.10	-0.25	0.10	0.13	3.589	0.038

Unfortunately, there was no papillary fill with this technique, which was expected. Gingival recession is usually observed in the presence of trauma and inflammation in individuals with thin phenotypes. GT assessment at the mandibular incisors is done using four methods. Since there is an augmentation of the gingiva, we can prevent a gingival recession. In this study, there was a statistically significant increase in GT after six months, along with an increase in KTW. Adequately attached gingiva enables patients to maintain oral hygiene, gives support to marginal gingiva, and helps to withstand the functional stresses of mastication. Also, it provides attachment for the movable alveolar mucosa for the action of the cheeks, lips, and tongue (Figure 2).

## Discussion

Accurate knowledge and understanding of how tissue responds to therapy are crucial for achieving a favorable cosmetic result in periodontal therapy, dental implants, and restorative procedures. One of the crucial factors affecting the success of aesthetic treatment is the gingival biotype, which has been drawing a lot of attention recently [3].

Developments in this field are primarily focusing on making PRF injectable by generating a liquid formulation of PRF devoid of anticoagulants and fibrin matrix, which would allow for a greater concentration of regeneration cells and a higher expression of growth factors. One of the advantages of MN is that it increases the epidermis' permeability and blood flow. This procedure makes it easier for topical drugs and growth factors to cross the stratum corneum and support the regeneration of collagen and elastin. A review by Esfahrood et al. found that gingival biotype significantly influences the results of periodontal therapy, root-covering surgeries, and implant placement [9]. Peixoto et al. found a positive correlation between the width of keratinized tissue, probing depth, and clinical attachment level [10]. Anderegg et al. found that there was less post-treatment gingival recession for tissue thickness >1 mm than for tissue thickness ≤1 mm [11]. Baldi et al. showed that robust gingival tissue was associated with a more favorable clinical result for root coverage than thin gingival tissue [12]. GT was influenced by the use of MN and the use of i-PRF, which could be due to the injury caused by MN, which results in collagen induction, and i-PRF contributes to faster healing by providing growth factors.

The gingival phenotype may be described in part by buccal-lingual GT, which, together with tooth movement direction, is thought to have a significant role in the development and progression of mucogingival abnormalities [13]. As nearby sedative infusions can advance capacity in the tissue and vasoconstrictor synthetics could impact the dissemination of i-PRF to be managed, an effective sedative was applied before the MN/i-PRF methods and GT assessment through trans-gingival testing [14]. Hakkinen et al.'s study showed that within 7-14 days, overall collagen synthesis rose throughout the wound-healing process [15]. Miron et al. analyzed PRF resorption and found that it took 7-11 days, whereas i-PRF growth factor release occurred on day 10 [16]. As a result, four courses of i-PRF and MN + i-PRF were administered separated by 10-day intervals.

It was observed in the study that there was an increase in KTW of the central incisors, lateral incisors, and canine of the lower anterior in the group receiving MN along with i-PRF at the end of six months, but it was not statistically significant. Statistically significant increases were seen in the GT of the central incisors, lateral incisors, and canine of the lower anterior in the group receiving MN along with i-PRF at the end of six months. This study examined a non-surgical method of augmenting gingival tissue. The properties of neoangiogenesis and neo-collagenases of MN had an additional effect on i-PRF in augmenting the gingival biotype. Due to the tight nature of the keratinized tissue, the i-PRF was injected solely into the apical mucogingival edge using a 27-gauge dental injector, which may be swapped with an insulin syringe, in order to minimize patient stress. There was no way to assess the dental arch's position due to missing teeth.

We can redefine the procedure by using a derma roller. With its help, we can also measure the number of microchannels in the mandibular anterior region where the procedure was done. Non-surgical gingival augmentation with MN has found favor with patients due to its less time-consuming and minimally invasive nature, causing less discomfort.

i-PRF has many advantages in the regeneration process, i.e., it is rich in platelets, leucocytes, and growth factors. It helps in higher fibroblast migration as well as higher expression of PDGF, transforming growth factor, and type-1 collagen. The three-dimensional fibrin matrix plays a key role in tissue repair. Fibrin acts as a scaffolding biological material for the agglomeration of adherent cells at the site of tissue healing. Also, fibrin is a carrier of growth factors in a well-controlled release system that sustains proper bioactivity over the healing period. The MN system helps increase the production of elastin fiber and collagen, causing neoangiogenesis.

The limitations were as follows: the injection of i-PRF was done only into the apical mucogingival margin due to the tight structure of the keratinized tissue; there was a lack of evaluation of the position of teeth in the dental arch; and very few studies have been done on MN for gingiva. Further research, clinical trials, and studies are required for further evaluation of the effectiveness of this technique.

## Conclusions

We described a novel method for enlarging the gums without resorting to invasive procedures like innervations. MN's neoangiogenesis impact contributed further to the thickening of the gingiva, whereas i-PRF contributed to enhanced growth factor release. Our findings endorse the clinical use of MN along with i-PRF; this is more comfortable for patients as it causes less pain and trauma.

## References

[1] Stellini E, Comuzzi L, Mazzocco F, Parente N, Gobbato L (2013). Relationships between different tooth shapes and patient's periodontal phenotype. J Periodontal Res.

[2] Jepsen S, Caton JG, Albandar JM (2018). Periodontal manifestations of systemic diseases and developmental and acquired conditions: consensus report of workgroup 3 of the 2017 World Workshop on the Classification of Periodontal and Peri-Implant Diseases and Conditions. J Periodontol.

[3] Abraham S, Deepak KT, Ranjith A, Preeja C, Archana V (2014). Gingival biotype and its clinical significance - a review. Saudi J Dent Res.

[4] Singh A, Yadav S (2016). Microneedling: advances and widening horizons. Indian Dermatol Online J.

[5] Batra P, Dawar A, Miglani S (2020). Microneedles and nanopatches-based delivery devices in dentistry. Discoveries (Craiova).

[6] Toffler M, Toscano N, Holtzclaw D, Del Corso M, Ehrenfest DM (2019). Introducing Choukroun’s platelet rich fibrin (PRF) to the reconstructive surgery milieu. J Implant Adv Clin Dent.

[7] Gollapudi M, Bajaj P, Oza RR (2022). Injectable platelet-rich fibrin - a revolution in periodontal regeneration. Cureus.

[8] Agrawal DR, Jaiswal PG (2020). Injectable platelet rich fibrin (i-PRF): a gem in dentistry. Int J Curr Res Rev.

[9] Esfahrood ZR, Kadkhodazadeh M, Talebi Ardakani MR (2013). Gingival biotype: a review. Gen Dent.

[10] Peixoto A, Marques TM, Correia A (2015). Gingival biotype characterization--a study in a Portuguese sample. Int J Esthet Dent.

[11] Anderegg CR, Metzler DG, Nicoll BK (1995). Gingiva thickness in guided tissue regeneration and associated recession at facial furcation defects. J Periodontol.

[12] Baldi C, Pini-Prato G, Pagliaro U, Nieri M, Saletta D, Muzzi L, Cortellini P (1999). Coronally advanced flap procedure for root coverage. Is flap thickness a relevant predictor to achieve root coverage? A 19-case series. J Periodontol.

[13] Miyasato M, Crigger M, Egelberg J (1977). Gingival condition in areas of minimal and appreciable width of keratinized gingiva. J Clin Periodontol.

[14] Kloukos D, Koukos G, Doulis I, Sculean A, Stavropoulos A, Katsaros C (2018). Gingival thickness assessment at the mandibular incisors with four methods: a cross-sectional study. J Periodontol.

[15] Hakkinen L, Larjava H, Koivisto L (2018). Granulation tissue formation and remodeling. Oral wound healing: Cell Biology and Clinical Management.

[16] Miron RJ, Fujioka-Kobayashi M, Hernandez M, Kandalam U, Zhang Y, Ghanaati S, Choukroun J (2017). Injectable platelet rich fibrin (i-PRF): opportunities in regenerative dentistry?. Clin Oral Investig.

